# Supplementary material for: Multivariate analysis of PET pharmacokinetic parameters improves inferential efficiency
Source: EJNMMI Phys. 2023 Mar 13;10:17. doi: 10.1186/s40658-023-00537-8 (PMC10008760; doi:10.1186/s40658-023-00537-8)
Supplement: Supplementary file 1 — Additional file 1. Supplementary Materials SX. [file 40658_2023_537_MOESM1_ESM.pdf]

Supplementary Materials

Contents

Supplementary Materials S1 : Prior Specification **2**

Global Intercepts 2

Individual deviations 3

Regional deviations 4

Residuals 5

Covariates 6

Supplementary Materials S2 : Simulation Parameters **7**

Global Intercepts 7

Regional Deviations 9

Covariance Matrices 10

Supplementary Materials S3 : TAC Simulation Additional Figures **17**

Supplementary Materials S4 : Comparison with SiMBA Additional Figures **20**

Supplementary Materials S5 : Correlated and Uncorrelated Data **22**

Supplementary Materials S6 : Parameter Simulation Additional Figures **27**

Supplementary Materials S7 : Parameter Simulation Additional Figures **28**

Supplementary Materials S1 : Prior Specification

Global Intercepts

Below are the priors defined for the global intercepts. Note that all priors are defined over the natural logarithms of the parameters.

Two-Tissue Compartment Model

[^{11}C]WAY100635

$$\begin{aligned}
\alpha_{\mathrm{K_1}} &\sim \mathrm{Normal}(-2.5\ ,\ 0.25) \\
\alpha_{\mathrm{V_{ND}}} &\sim \mathrm{Normal}(-1.0\ ,\ 0.25) \\
\alpha_{\mathrm{BP_P}} &\sim \mathrm{Normal}(\ \ 1.0\ ,\ 0.25) \\
\alpha_{\mathrm{k_4}} &\sim \mathrm{Normal}(-4.0\ ,\ 0.25)
\end{aligned}$$

The priors for $\alpha_{\mathrm{K_1}}$ and $\alpha_{\mathrm{BP_P}}$ are defined for the dorsolateral prefrontal cortex as the reference level of the dummy variable.

[^{11}C]ABP688

$$\begin{aligned}
\alpha_{\mathrm{K_1}} &\sim \mathrm{Normal}(-2.0\ ,\ 0.25) \\
\alpha_{\mathrm{V_{ND}}} &\sim \mathrm{Normal}(-0.5\ ,\ 0.5) \\
\alpha_{\mathrm{BP_P}} &\sim \mathrm{Normal}(\ \ 0.5\ ,\ 0.5) \\
\alpha_{\mathrm{k_4}} &\sim \mathrm{Normal}(-2.5\ ,\ 0.5)
\end{aligned}$$

The priors for $\alpha_{\mathrm{K_1}}$ and $\alpha_{\mathrm{BP_P}}$ are defined for the dorsolateral prefrontal cortex as the reference level of the dummy variable.

One-Tissue Compartment Model

[^{11}C]DASB

$$\begin{aligned}
\alpha_{\mathrm{K_1}} &\sim \mathrm{Normal}(-1\ ,\ 0.25) \\
\alpha_{\mathrm{V_T}} &\sim \mathrm{Normal}(2\ ,\ 0.25)
\end{aligned}$$

The priors for $\alpha_{\mathrm{K_1}}$ and $\alpha_{\mathrm{V_T}}$ are defined for the anterior cingulate cortex as the reference level of the dummy variable.

[^{11}C]GR103545

$$\begin{aligned}
\alpha_{\mathrm{K_1}} &\sim \mathrm{Normal}(-2.0\ ,\ 0.25) \\
\alpha_{\mathrm{V_T}} &\sim \mathrm{Normal}(3\ ,\ 0.5)
\end{aligned}$$

The priors for $\alpha_{\mathrm{K_1}}$ and $\alpha_{\mathrm{V_T}}$ are defined for the dorsolateral prefrontal cortex as the reference level of the dummy variable.

Simplified Reference Tissue Model

[11C]DASB

$$\begin{aligned}
\alpha_{\mathrm{R_1}} &\sim \mathrm{Normal}(0\,,\ 0.25)\\
\alpha_{\mathrm{k'_2}} &\sim \mathrm{Normal}(-3\,,\ 0.25)\\
\alpha_{\mathrm{BP_{ND}}} &\sim \mathrm{Normal}(-1\,,\ 0.25)
\end{aligned}$$

The priors for $\alpha_{\mathrm{R_1}}$ and $\alpha_{\mathrm{BP_{ND}}}$ are defined for the anterior cingulate cortex as the reference level of the dummy variable.

[11C]WAY100635

$$\begin{aligned}
\alpha_{\mathrm{R_1}} &\sim \mathrm{Normal}(0\,,\ 0.25)\\
\alpha_{\mathrm{k'_2}} &\sim \mathrm{Normal}(-2.3\,,\ 0.25)\\
\alpha_{\mathrm{BP_{ND}}} &\sim \mathrm{Normal}(1.5\,,\ 0.25)
\end{aligned}$$

The priors for $\alpha_{\mathrm{R_1}}$ and $\alpha_{\mathrm{BP_{ND}}}$ are defined for the dorsolateral prefrontal cortex as the reference level of the dummy variable.

Individual deviations

Differences between individuals were defined by specifying the primary pharmacokinetic parameters in one variance-covariance matrix.

Two-Tissue Compartment Model

For both [11C]WAY100635 and [11C]ABP688, we used the same priors.

$$\begin{bmatrix}\tau_{\mathrm{K_1}}\\ \tau_{\mathrm{V_{ND}}}\\ \tau_{\mathrm{BP_{ND}}}\\ \tau_{\mathrm{k_4}}\end{bmatrix} \sim \mathrm{MVNormal}\left(\begin{bmatrix}0\\0\\0\\0\end{bmatrix}, \mathbf{\Sigma}_{\mathrm{Subject}}\right)$$

$$\Sigma_{\mathrm{Subject}} = \begin{bmatrix}\sigma_{\mathrm{K_1}} & 0 & 0\\ 0 & \ddots & 0\\ 0 & 0 & \sigma_{\mathrm{k_4}}\end{bmatrix} \mathbf{R}_{\mathrm{Subject}} \begin{bmatrix}\sigma_{\mathrm{K_1}} & 0 & 0\\ 0 & \ddots & 0\\ 0 & 0 & \sigma_{\mathrm{k_4}}\end{bmatrix}$$

$$\begin{aligned}
\sigma_{\mathrm{K_1}} &\sim \text{Half-Normal}(0\,,\ 0.3)\\
\sigma_{\mathrm{V_{ND}}} &\sim \text{Half-Normal}(0\,,\ 0.3)\\
\sigma_{\mathrm{BP_{ND}}} &\sim \text{Half-Normal}(0\,,\ 0.3)\\
\sigma_{\mathrm{k_4}} &\sim \text{Half-Normal}(0\,,\ 0.3)\\
\mathbf{R}_{\mathrm{Subject}} &\sim \mathrm{LKJ}(1)
\end{aligned}$$

One-Tissue Compartment Model

For both [^{11}C]DASB and [^{11}C]GR103545, we used the same priors.

$$
\begin{aligned}
\begin{bmatrix} \tau_{\mathrm{K_1}} \\ \tau_{\mathrm{V_T}} \end{bmatrix} &\sim \mathrm{MVNormal}\left(\begin{bmatrix} 0 \\ 0 \end{bmatrix}, \mathbf{\Sigma}_{\mathrm{Subject}} \right) \\
\Sigma_{\mathrm{Subject}} &= \begin{bmatrix} \sigma_{\mathrm{K_1}} & 0 \\ 0 & \sigma_{\mathrm{V_T}} \end{bmatrix} \mathbf{R}_{\mathrm{Subject}} \begin{bmatrix} \sigma_{\mathrm{K_1}} & 0 \\ 0 & \sigma_{\mathrm{V_T}} \end{bmatrix} \\
\\
\sigma_{\mathrm{K_1}} &\sim \text{Half-Normal}(0\,,\ 0.3) \\
\sigma_{\mathrm{V_T}} &\sim \text{Half-Normal}(0\,,\ 0.3) \\
\mathbf{R}_{\mathrm{Subject}} &\sim \mathrm{LKJ}(1)
\end{aligned}
$$

Simplified Reference Tissue Model

For both [^{11}C]DASB and [^{11}C]WAY100635, we used the same priors.

$$
\begin{aligned}
\begin{bmatrix} \tau_{\mathrm{R_1}} \\ \tau_{\mathrm{k_2'}} \\ \tau_{\mathrm{BP_{ND}}} \end{bmatrix} &\sim \mathrm{MVNormal}\left(\begin{bmatrix} 0 \\ 0 \\ 0 \end{bmatrix}, \mathbf{\Sigma}_{\mathrm{Subject}} \right) \\
\Sigma_{\mathrm{Subject}} &= \begin{bmatrix} \sigma_{\mathrm{R_1}} & 0 & 0 \\ 0 & \sigma_{\mathrm{k_2'}} & 0 \\ 0 & 0 & \sigma_{\mathrm{BP_{ND}}} \end{bmatrix} \mathbf{R}_{\mathrm{Subject}} \begin{bmatrix} \sigma_{\mathrm{R_1}} & 0 & 0 \\ 0 & \sigma_{\mathrm{k_2'}} & 0 \\ 0 & 0 & \sigma_{\mathrm{BP_{ND}}} \end{bmatrix} \\
\\
\sigma_{\mathrm{R_1}} &\sim \text{Half-Normal}(0\,,\ 0.3) \\
\sigma_{\mathrm{k_2'}} &\sim \text{Half-Normal}(0\,,\ 0.3) \\
\sigma_{\mathrm{BP_{ND}}} &\sim \text{Half-Normal}(0\,,\ 0.3) \\
\mathbf{R}_{\mathrm{Subject}} &\sim \mathrm{LKJ}(1)
\end{aligned}
$$

Regional deviations

For $\log\mathrm{BP_{ND}}$ and $\log\mathrm{K_1}$, regional differences were defined as unpooled effects using a dummy (indicator) variable defined with reference to the dorsolateral prefrontal cortex. For simplicity, all regional differences (with the exception of [^{11}C]DASB) were defined as zero-centred regularising priors with the same SD.

$$
\begin{aligned}
\upsilon_{\mathrm{j,K_1}} &\sim \mathrm{Normal}(0\,,\ 0.3) \\
\upsilon_{\mathrm{j,BP_{ND}}} &\sim \mathrm{Normal}(0\,,\ 0.3)
\end{aligned}
$$

For [^{11}C]DASB, the same standard deviations were used, but with altered means for regions whose mean binding values were very different from the other regions. For SRTM, the mean values for $$ were defined as follows:

- Insula: 0.5

- Amygdala, dorsal putamen, thalamus, ventral striatum: 1
- Midbrain: 1.5

For the 1TC, the means were defined as follows:

- Dorsal putamen, ventral striatum: 0.5
- Midbrain: 1

For the remaining parameters, regional differences were defined as pooled variables, arising from a common distribution

$$\begin{aligned}
\begin{bmatrix} v \\ \vdots \end{bmatrix} &\sim \text{MVNormal}\left(\begin{bmatrix} 0 \\ \vdots \end{bmatrix}, \boldsymbol{\Sigma}_{\text{Region}}\right) \\
\Sigma_{\text{Region}} &= \begin{bmatrix} \sigma \\ \vdots \end{bmatrix} \mathbf{R}_{\text{Region}} \begin{bmatrix} \sigma \\ \vdots \end{bmatrix} \\
\sigma &\sim \text{Half-Normal}(0\,,\ 0.1) \\
\mathbf{R}_{\text{Region}} &\sim \text{LKJ}(2)
\end{aligned}$$

Residuals

Two-Tissue Compartment Model

For both [^{11}C]WAY100635 and [^{11}C]ABP688, we used the same priors.

$$\begin{aligned}
\begin{bmatrix} \epsilon_{\text{K}_1} \\ \epsilon_{\text{V}_{\text{ND}}} \\ \epsilon_{\text{BP}_{\text{ND}}} \\ \epsilon_{\text{k}_4} \end{bmatrix} &\sim \text{MVNormal}\left(\begin{bmatrix} 0 \\ 0 \\ 0 \\ 0 \end{bmatrix}, \boldsymbol{\Sigma}_{\text{Subject}}\right) \\
\Sigma_{\text{Subject}} &= \begin{bmatrix} \sigma_{\text{K}_1} & 0 & 0 \\ 0 & \ddots & 0 \\ 0 & 0 & \sigma_{\text{k}_4} \end{bmatrix} \mathbf{R}_{\text{Subject}} \begin{bmatrix} \sigma_{\text{K}_1} & 0 & 0 \\ 0 & \ddots & 0 \\ 0 & 0 & \sigma_{\text{k}_4} \end{bmatrix} \\
\sigma_{\text{K}_1} &\sim \text{Half-Normal}(0\,,\ 0.1) \\
\sigma_{\text{V}_{\text{ND}}} &\sim \text{Half-Normal}(0\,,\ 0.1) \\
\sigma_{\text{BP}_{\text{ND}}} &\sim \text{Half-Normal}(0\,,\ 0.1) \\
\sigma_{\text{k}_4} &\sim \text{Half-Normal}(0\,,\ 0.1) \\
\mathbf{R}_{\text{Subject}} &\sim \text{LKJ}(2)
\end{aligned}$$

One-Tissue Compartment Model

For both [^{11}C]DASB and [^{11}C]GR103545, we used the same priors.

$$
\begin{bmatrix} \epsilon_{\mathrm{K_1}} \\ \epsilon_{\mathrm{V_T}} \end{bmatrix} \sim \mathrm{MVNormal}\left(\begin{bmatrix} 0 \\ 0 \end{bmatrix}, \mathbf{\Sigma}_{\mathrm{Subject}} \right)
$$

$$
\Sigma_{\mathrm{Subject}} = \begin{bmatrix} \sigma_{\mathrm{K_1}} & 0 \\ 0 & \sigma_{\mathrm{V_T}} \end{bmatrix} \mathbf{R}_{\mathrm{Subject}} \begin{bmatrix} \sigma_{\mathrm{K_1}} & 0 \\ 0 & \sigma_{\mathrm{V_T}} \end{bmatrix}
$$

$$
\begin{aligned}
\sigma_{\mathrm{K_1}} &\sim \text{Half-Normal}(0\,,\ 0.1) \\
\sigma_{\mathrm{V_T}} &\sim \text{Half-Normal}(0\,,\ 0.1) \\
\mathbf{R}_{\mathrm{Subject}} &\sim \mathrm{LKJ}(2)
\end{aligned}
$$

Simplified Reference Tissue Model

For both [^{11}C]DASB and [^{11}C]WAY100635, we used the same priors.

$$
\begin{bmatrix} \epsilon_{\mathrm{R_1}} \\ \epsilon_{\mathrm{k_2'}} \\ \epsilon_{\mathrm{BP_{ND}}} \end{bmatrix} \sim \mathrm{MVNormal}\left(\begin{bmatrix} 0 \\ 0 \\ 0 \end{bmatrix}, \mathbf{\Sigma}_{\mathrm{Subject}} \right)
$$

$$
\Sigma_{\mathrm{Subject}} = \begin{bmatrix} \sigma_{\mathrm{R_1}} & 0 & 0 \\ 0 & \sigma_{\mathrm{k_2'}} & 0 \\ 0 & 0 & \sigma_{\mathrm{BP_{ND}}} \end{bmatrix} \mathbf{R}_{\mathrm{Subject}} \begin{bmatrix} \sigma_{\mathrm{R_1}} & 0 & 0 \\ 0 & \sigma_{\mathrm{k_2'}} & 0 \\ 0 & 0 & \sigma_{\mathrm{BP_{ND}}} \end{bmatrix}
$$

$$
\begin{aligned}
\sigma_{\mathrm{R_1}} &\sim \text{Half-Normal}(0\,,\ 0.1) \\
\sigma_{\mathrm{k_2'}} &\sim \text{Half-Normal}(0\,,\ 0.1) \\
\sigma_{\mathrm{BP_{ND}}} &\sim \text{Half-Normal}(0\,,\ 0.1) \\
\mathbf{R}_{\mathrm{Subject}} &\sim \mathrm{LKJ}(2)
\end{aligned}
$$

Covariates

For the simulations, there was only one additional covariate for group effects on the binding parameter. For this, we used a zero-centred regularising prior. This prior conservatively assumes that no difference is most likely, and assigns 95% of its probability between differences of -48% and 48% differences between groups.

$$
\beta_{\mathrm{Group}} \sim \mathrm{Normal}(\,0\,,\ 0.2\,)
$$

Supplementary Materials S2 : Simulation Parameters

Below are presented the values of the parameters described in the section above in order to simulate new data. We used the posterior mean values estimated using empirical data for each application.

Global Intercepts

Two-Tissue Compartment Model

[^{11}C]WAY100635

Parameter	Mean
logK1	-2.33
logBPnd	1.75
logVnd	-0.62
logk4	-3.84
logvB	-3.63
logsigma	-4.96

[^{11}C]ABP688

Parameter	Mean
logK1	-1.95
logBPnd	0.86
logVnd	-0.33
logk4	-2.57
logvB	-3.26
logsigma	-5.08

One-Tissue Compartment Model

[^{11}C]DASB

Parameter	Mean
logK1	-0.76
logVt	2.73

[^{11}C]GR103545

Parameter	Mean
logK1	-1.87
logVt	3.05

Simplified Reference Tissue Model

[^{11}C]DASB

Parameter	Mean
logR1	-0.08
logk2prime	-3.06
logBPnd	-0.86

[^{11}C]WAY100635

Parameter	Mean
logR1	0.23
logk2prime	-2.52
logBPnd	1.60

Regional Deviations

Regional deviations are the deviations from the global intercept for each parameter in each region. The values will be zero for the dummy variable when fixed effects were used by definition.

Two-Tissue Compartment Model

[^{11}C]WAY100635

Region	logK1	logBPnd	logVnd	logk4	logvB	logsigma
DLPFC	0.00	0.00	-0.09	0.17	-0.07	-0.30
MPFC	-0.01	0.03	-0.06	0.14	0.06	-0.23
Hippocampus	-0.22	0.18	0.29	-0.22	-0.07	0.00
Amygdala	-0.33	0.10	0.01	-0.08	0.00	0.12
Parahippocampus	-0.27	0.00	0.36	0.08	0.02	-0.10
Insula	-0.04	0.36	0.01	0.01	0.17	-0.06
ACC	-0.04	0.15	-0.05	0.09	0.15	-0.11
PCC	-0.02	-0.01	-0.10	0.12	0.13	-0.01
DRN	-0.30	-0.03	-0.31	-0.30	-0.40	0.69

[^{11}C]ABP688

Region	logK1	logBPnd	logVnd	logk4	logvB	logsigma
DLPFC	0.00	0.00	-0.05	-0.01	0.01	-0.32
Amygdala	-0.26	-0.04	-0.04	-0.06	-0.04	0.16
Hippocampus	-0.26	0.01	-0.12	-0.01	-0.08	-0.18
MPFC	0.01	0.00	0.02	0.05	0.08	-0.15
Dorsal Putamen	0.21	-0.13	0.09	-0.02	-0.04	0.04
Ventral Striatum	0.06	0.02	0.07	-0.02	-0.08	0.36
Insula	0.05	0.18	-0.01	0.00	0.05	-0.05
PCC	0.09	0.12	-0.05	0.01	0.05	0.10
ACC	0.08	0.11	0.08	0.06	0.05	0.02

One-Tissue Compartment Model

[^{11}C]DASB

Region	logK1	logVt
ACC	0.00	0.00
Dorsal Putamen	0.11	0.49
Ventral Striatum	0.01	0.63
Amygdala	-0.24	0.51
Thalamus	0.12	0.43
Hippocampus	-0.21	0.04
PCC	0.04	0.00
Insula	0.01	0.17
Midbrain	-0.19	0.84

[^{11}C]GR103545

Region	logK1	logVt
DLPFC	0.00	0.00
Amygdala	-0.51	0.15
Dorsal Putamen	-0.09	-0.29
Hippocampus	-0.33	-0.65
Insula	-0.19	0.20
MPFC	-0.10	0.08
Parahippocampus	-0.48	-0.13
DRN	-0.35	-0.88
Ventral Striatum	-0.20	0.16

Simplified Reference Tissue Model

[^{11}C]DASB

Region	logR1	logk2prime	logBPnd
ACC	0.00	-0.16	0.00
Dorsal Putamen	0.05	0.21	1.06
Ventral Striatum	-0.04	0.17	1.28
Amygdala	-0.26	0.07	1.11
Thalamus	0.08	0.13	0.97
Hippocampus	-0.17	-0.29	0.26
PCC	0.05	-0.24	0.05
Insula	-0.01	0.01	0.45
Midbrain	-0.21	0.07	1.63

[^{11}C]WAY100635

Region	logR1	logk2prime	logBPnd
DLPFC	0.00	0.00	0.00
MPFC	0.02	-0.01	0.07
Hippocampus	-0.11	0.10	0.47
Amygdala	-0.19	0.02	0.14
Parahippocampus	-0.13	0.20	0.41
Insula	0.02	0.07	0.42
ACC	0.02	0.00	0.19
PCC	0.02	-0.09	0.01
DRN	-0.27	-0.34	-0.24

Covariance Matrices

For the covariance matrices, the correlations and standard deviations have been separated for the purposes of visualisation and interpretation. The correlation matrices are shown above, with the relevant standard deviations presented in the table below. For the 2TC, we show the correlation matrix for the individual variation and region-within-individual variation as these parameters are estimated using SiMBA-derived parameters. For the 1TC and SRTM, we show the PuMBA-derived correlation matrices representing individual (τ) variation and residual (ϵ) variation.

Two-Tissue Compartment Model

[^{11}C]WAY100635. Note that these parameters are generated using SiMBA.

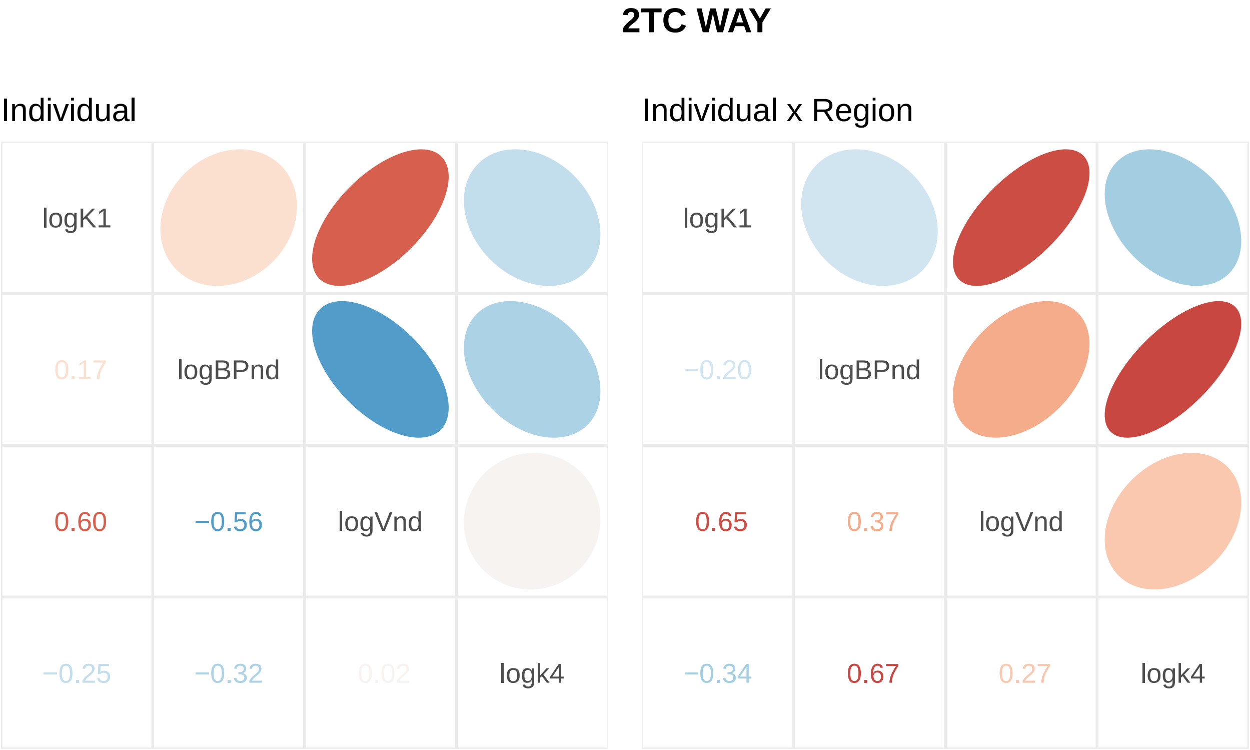

Standard Deviations

Grouping	logK1	logBPnd	logVnd	logk4
Individual	0.271	0.329	0.392	0.119
Individual x Region	0.053	0.037	0.054	0.016

[^{11}C]ABP688. Note that these parameters are generated using SiMBA.

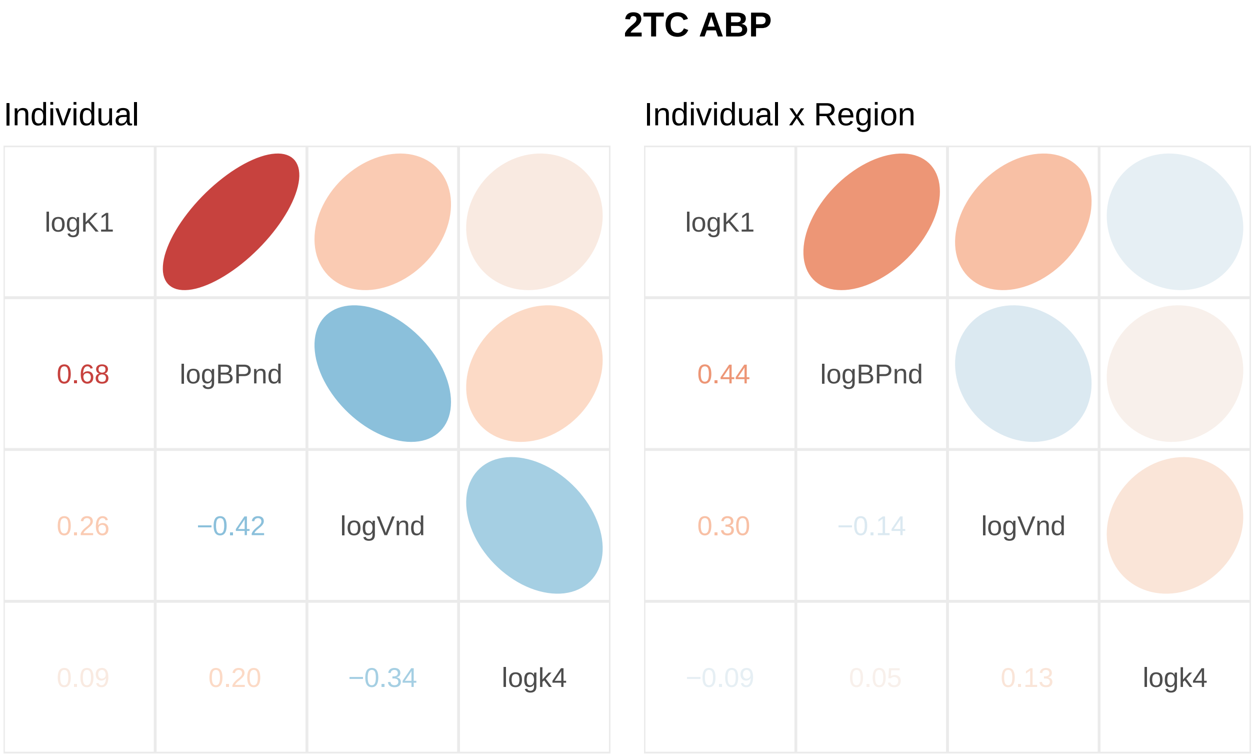

Standard Deviations

Grouping	logK1	logBPnd	logVnd	logk4
Individual	0.284	0.344	0.235	0.258
Individual x Region	0.038	0.035	0.032	0.032

One-Tissue Compartment Model

[^{11}C]DASB. Note that these parameters are generated using PuMBA.

1TC DASB

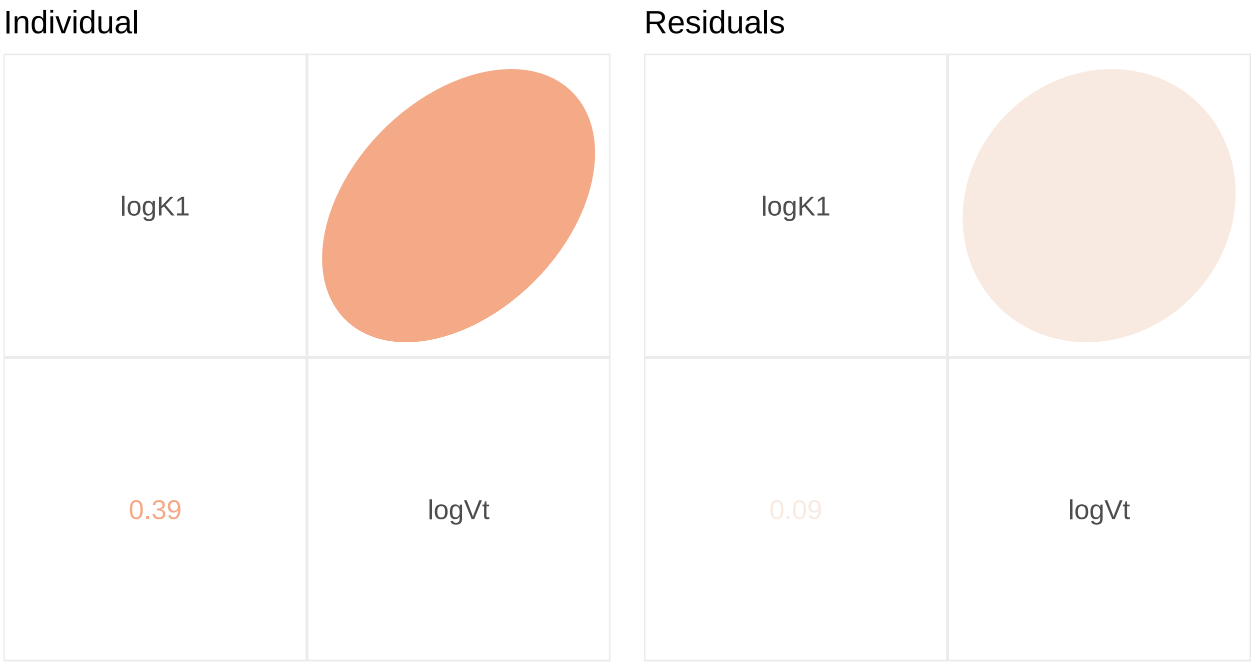

Standard Deviations

Grouping	logK1	logVt
Individual	0.208	0.203
Residual	0.064	0.066

[^{11}C]GR103545. Note that these parameters are generated using PuMBA.

1TC GR

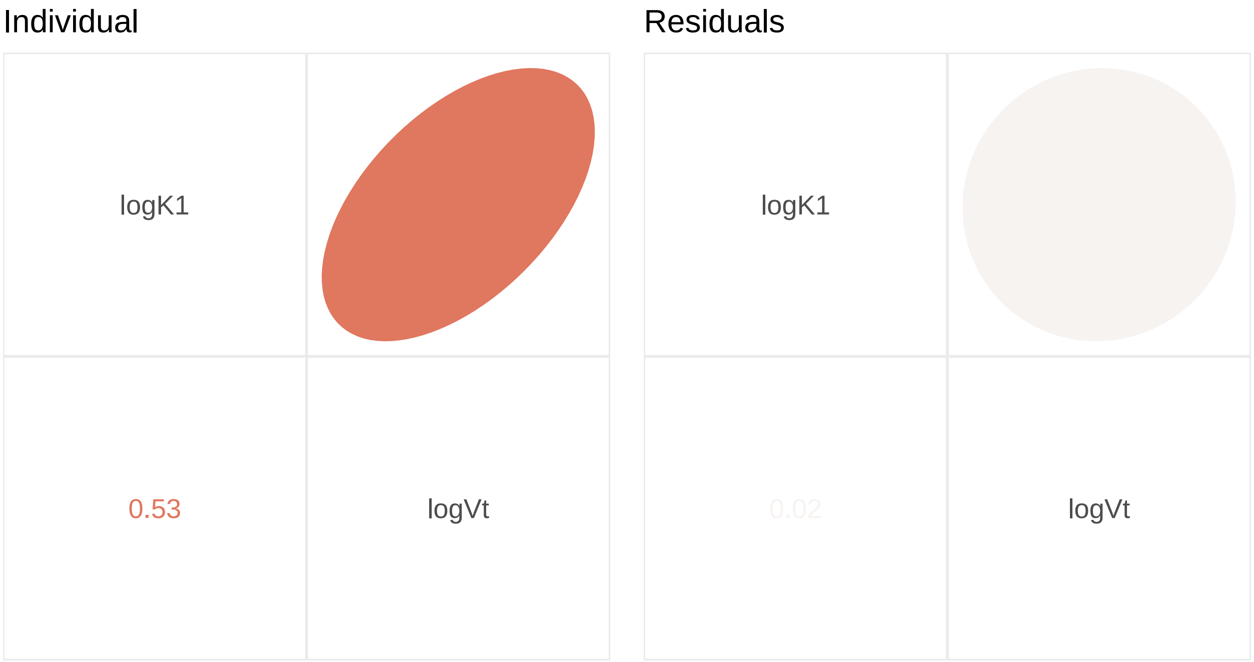

Standard Deviations

Grouping	logK1	logVt
Individual	0.266	0.312
Residual	0.041	0.103

Simplified Reference Tissue Model

[^{11}C]DASB. Note that these parameters are generated using PuMBA.

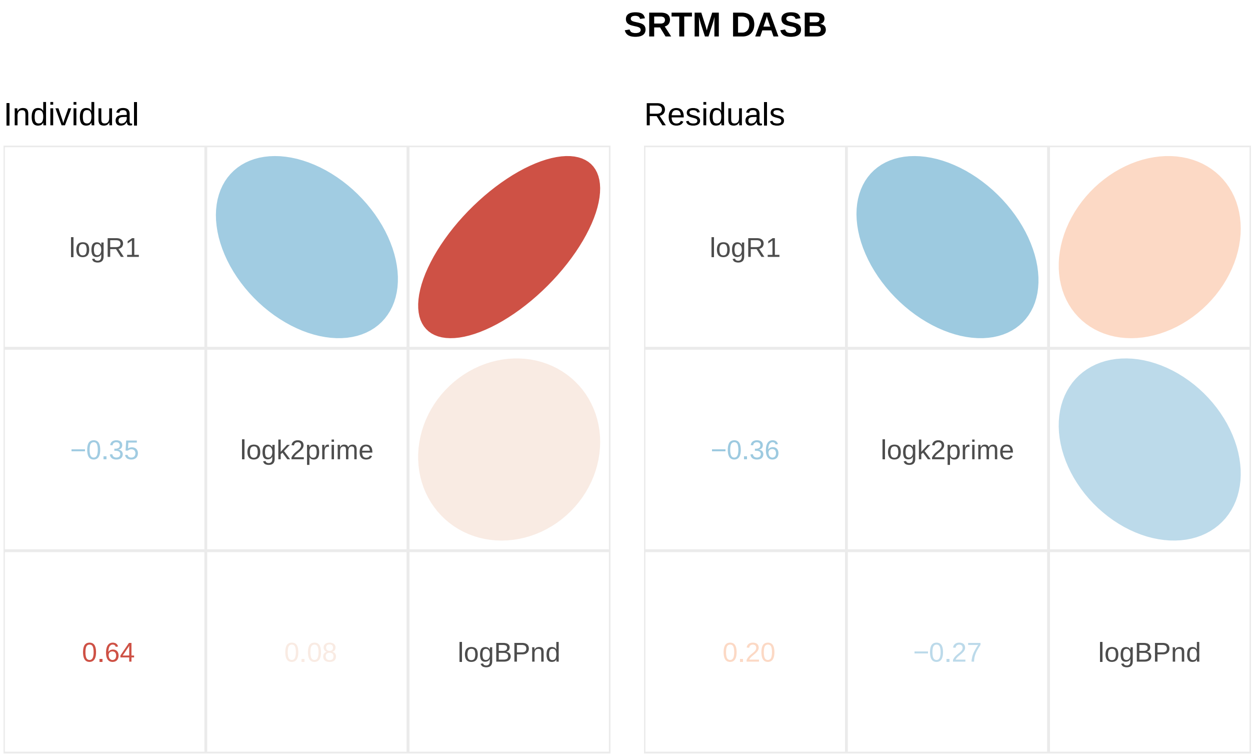

Standard Deviations

**Grouping**	**logR1**	**logk2prime**	**logBPnd**
Individual	0.088	0.190	0.202
Region	NA	0.172	NA
Residual	0.068	0.133	0.176

$[^{11}C]$WAY100635. Note that these parameters are generated using PuMBA.

SRTM WAY

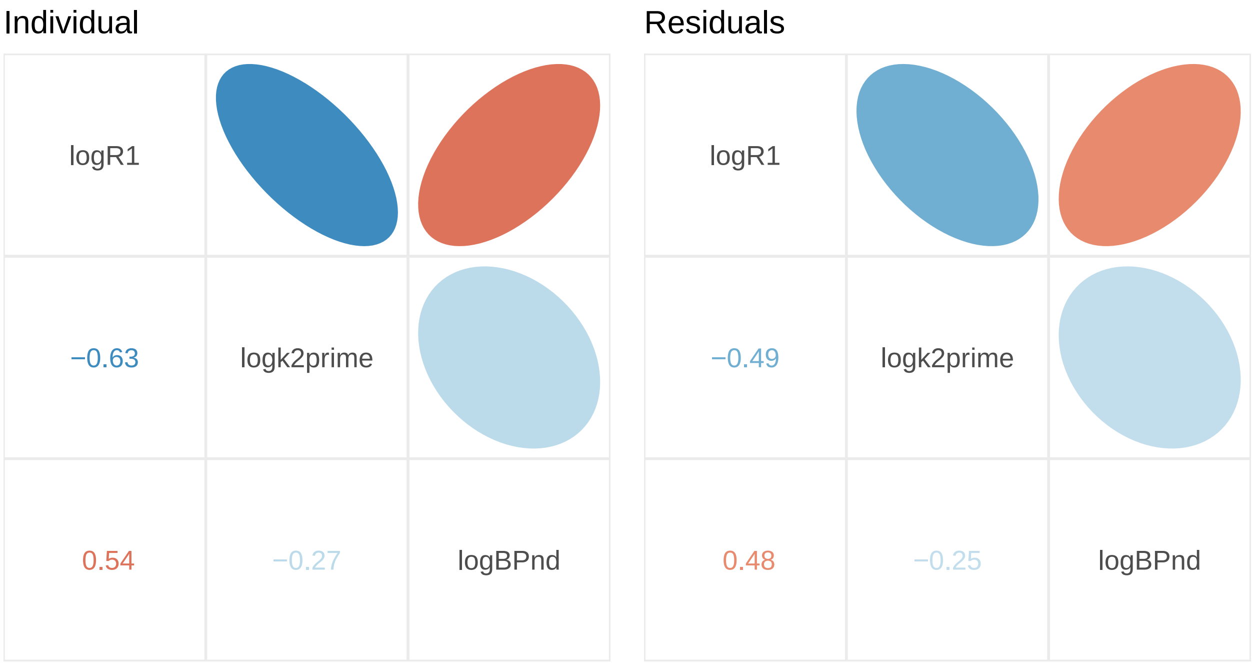

Standard Deviations

Grouping	logR1	logk2prime	logBPnd
Individual	0.123	0.142	0.230
Region	NA	0.149	NA
Residual	0.059	0.103	0.127

Supplementary Materials S3 : TAC Simulation Additional Figures

Here we show the additional figures regarding the outcomes of the TAC simulations, representing the false positive rate, the mean standard error, the standard deviation and the bias. Please refer to the manuscript for how each of these metrics are defined. Note that the bias is presented relative to the standard deviation of the group differences across the simulated datasets.

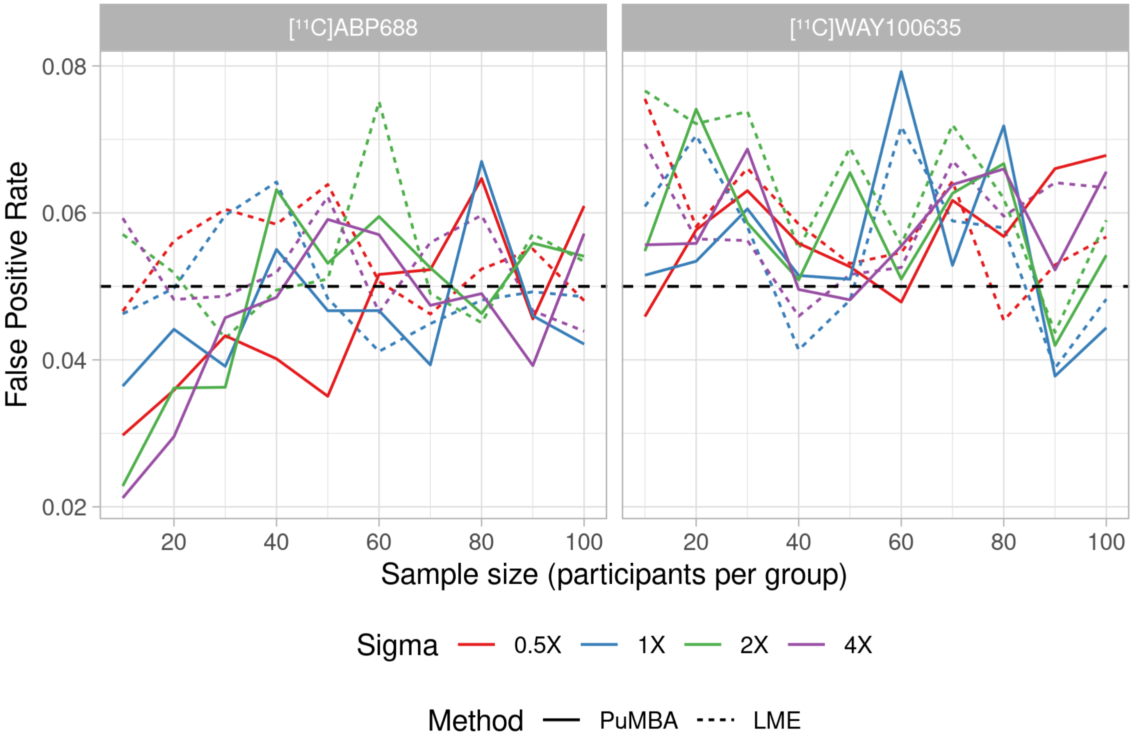

Figure 1: False positive rate across simulated datasets.

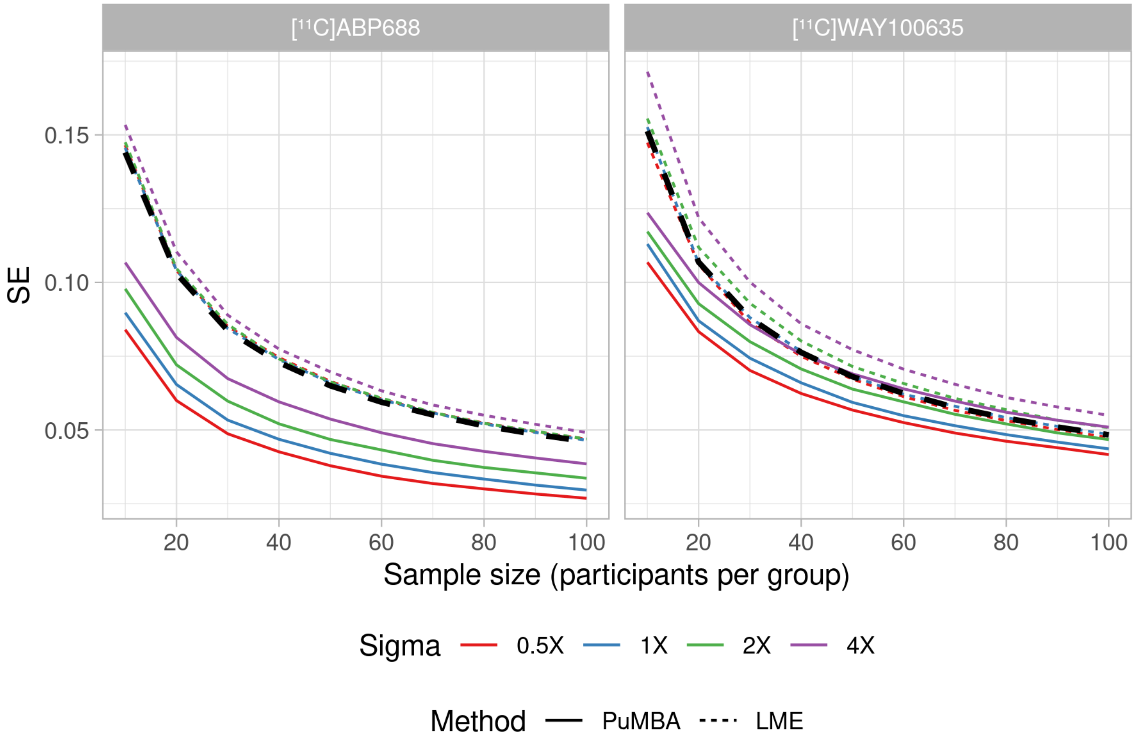

Figure 2: Mean standard error across simulated datasets.

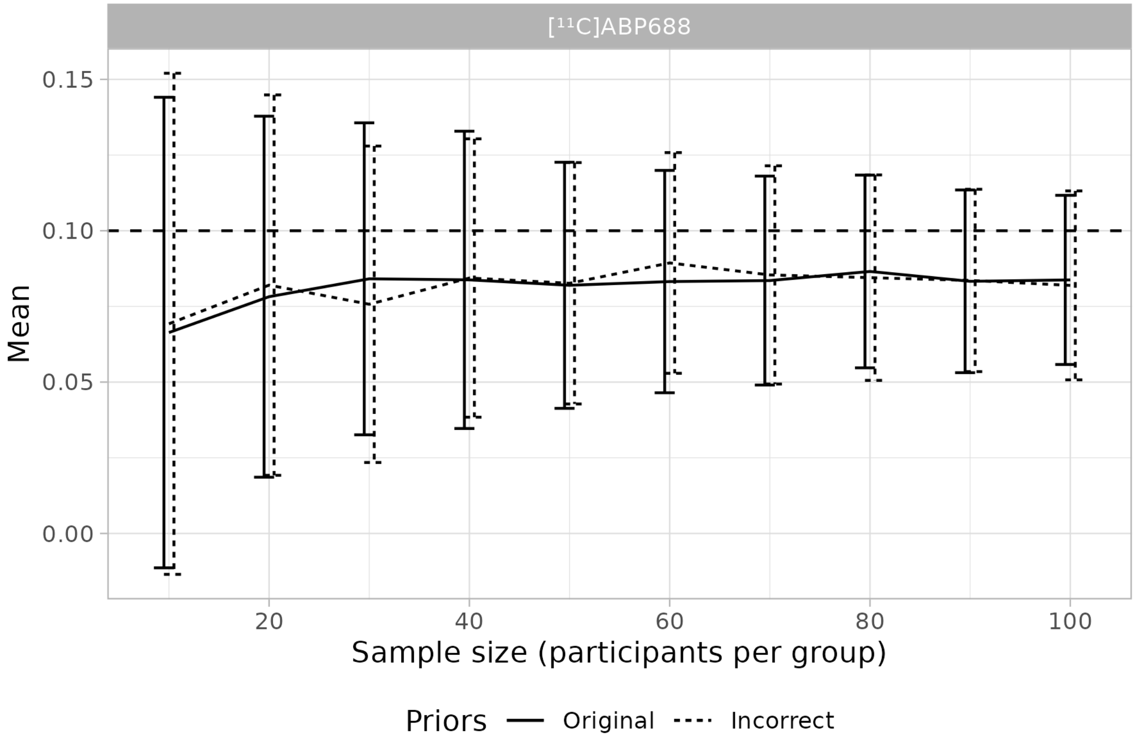

Figure 3: Mean estimated differences across simulated datasets.

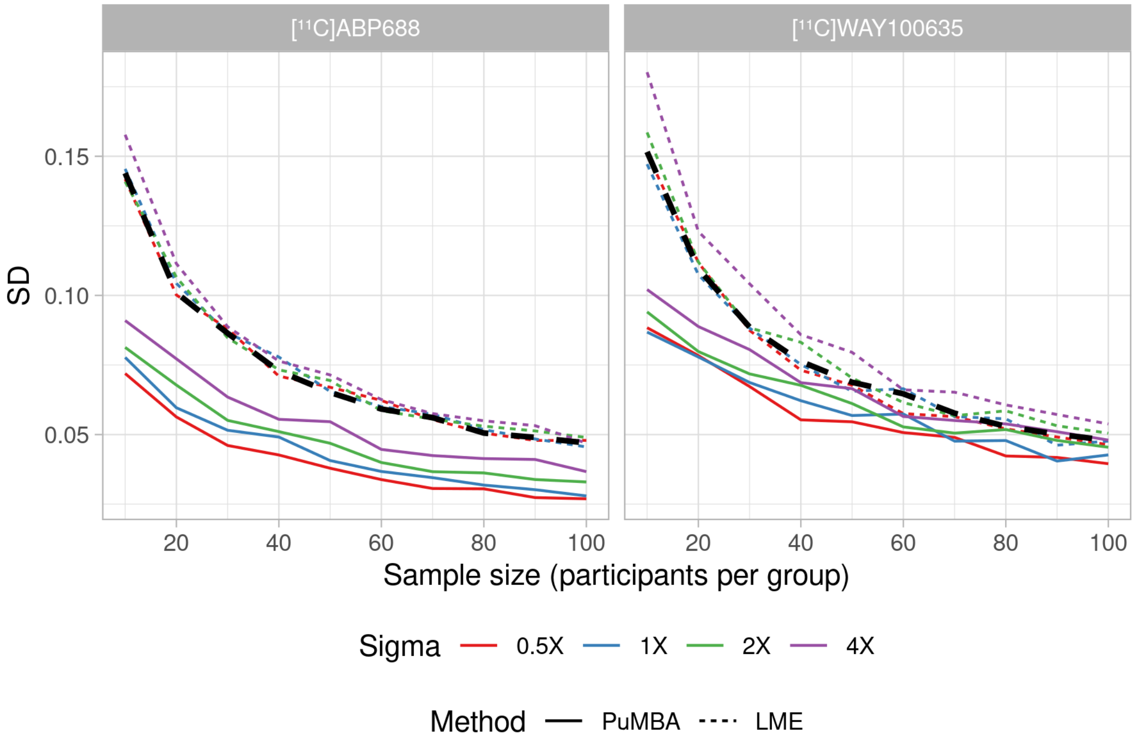

Figure 4: Standard deviation of estimated differences across simulated datasets.

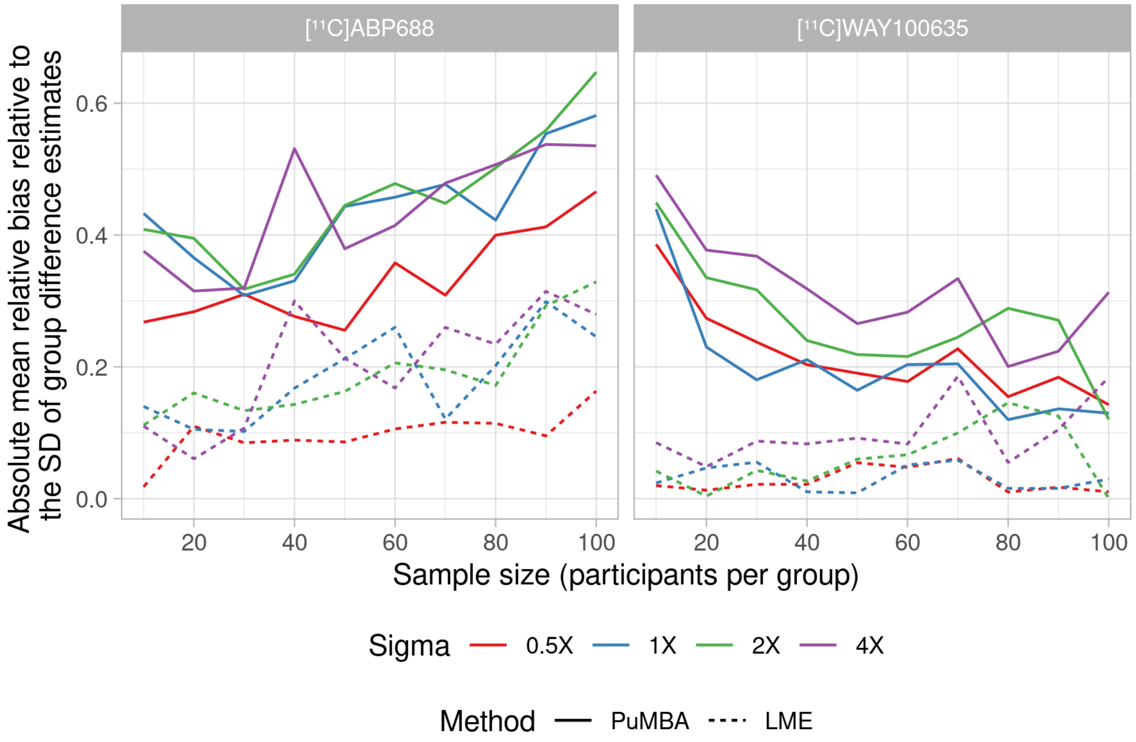

Figure 5: Bias of the estimated group difference estimates relative to the SD of estimated group differences across simulated datasets.

Supplementary Materials S4 : Comparison with SiMBA Additional Figures

Here we show additional outcomes from the TAC simulations in which PuMBA was compared with SiMBA. These figures show the false positive rate and standard deviation of estimates. Please refer to the manuscript for how each of these metrics are defined.

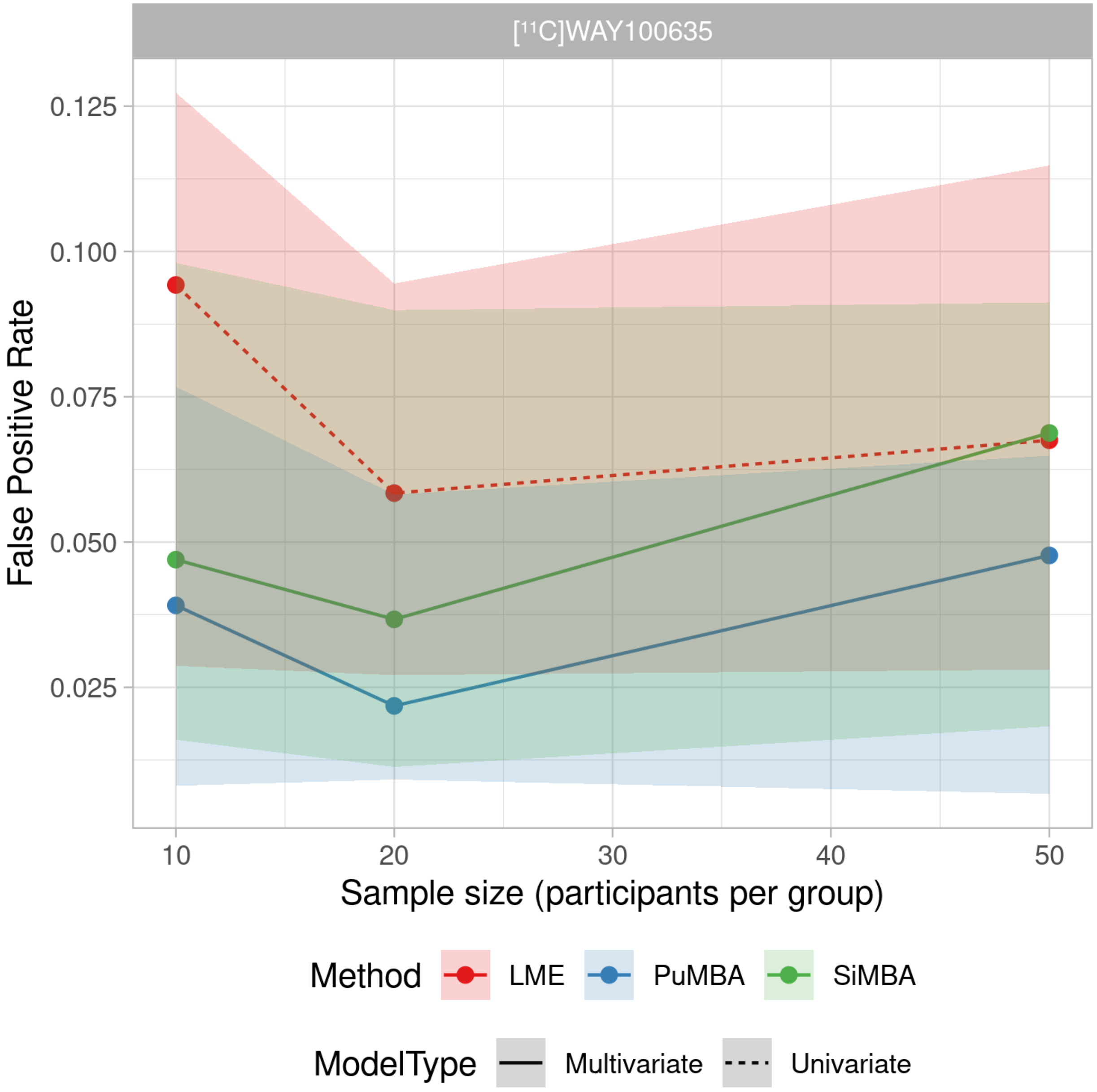

Figure 6: False positive rate across simulated datasets.

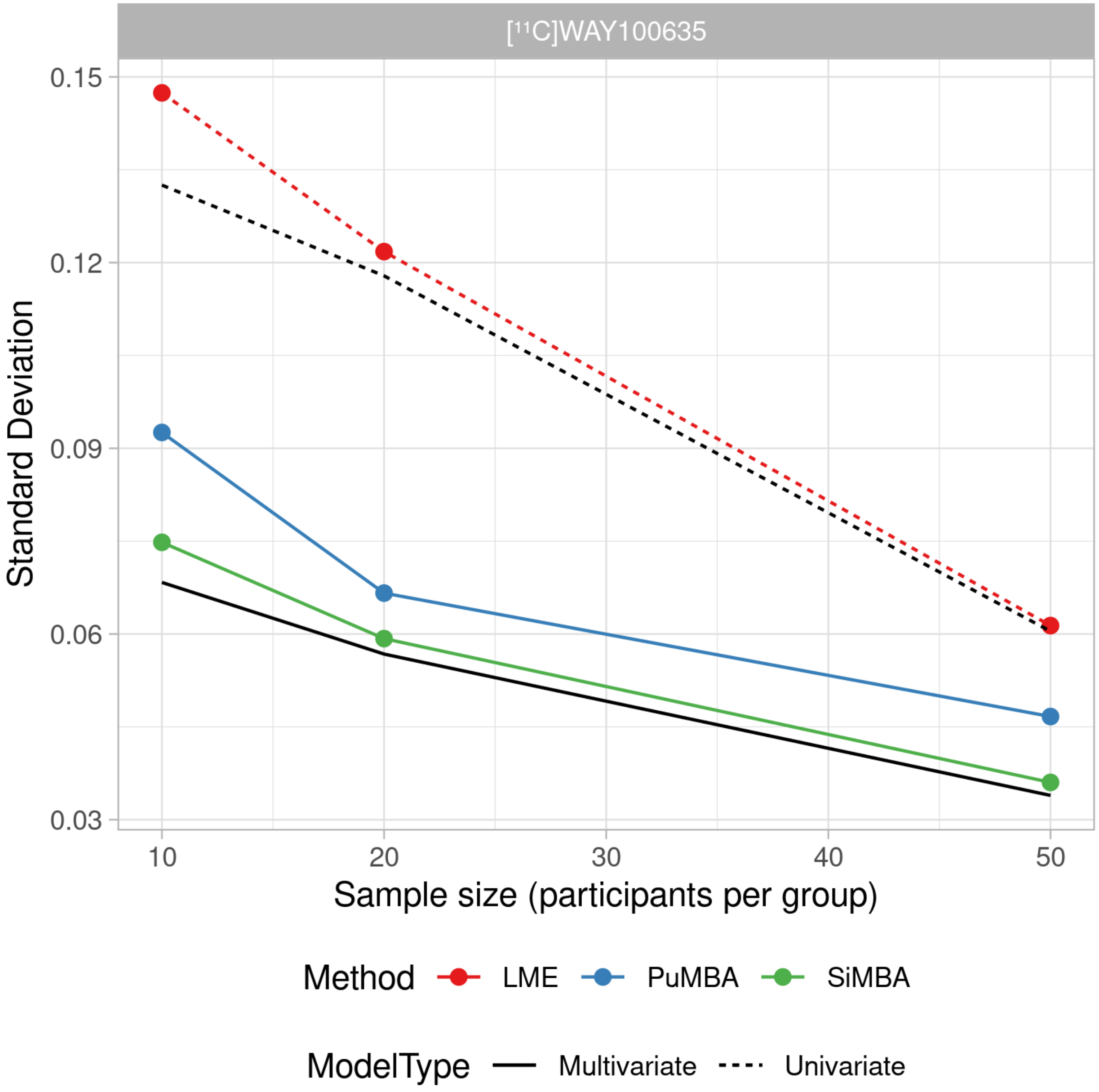

Figure 7: Standard deviation of estimated differences across simulated datasets.

Supplementary Materials S5 : Correlated and Uncorrelated Data

To assess correlation matrix recovery, we simulated both both parameters as well as TACs with the same mean and variance for all parameters, but using univariate instead of multivariate distributions, i.e. with no correlation structure between the parameters, for the individual (τ) and residual (ϵ) distributions in the parameter simulations, and for the individual and individual $\times$ region distributions for the TAC simulations using SiMBA. We generated 500 simulated datasets for each condition.

For the TAC simulations, residual correlation matrices for each tracer were very similar for correlated and uncorrelated data. This suggests that the correlations in the residual variation (ϵ) are primarily attributable to errors introduced during TAC fitting using NLS to generate the PK parameters owing to imperfect identifiability. Furthermore, comparing sample sizes of n=20, n=50 and n=100 per group, we observe broadly similar results, but with increased sample-to-sample variation in the smaller samples, suggesting that the poor parameter recovery is more attributable to NLS TAC fitting than to insufficient sample sizes. These residual correlation matrices differed greatly between tracers, suggesting that the nature of the imperfect identifiability is tracer-dependent.

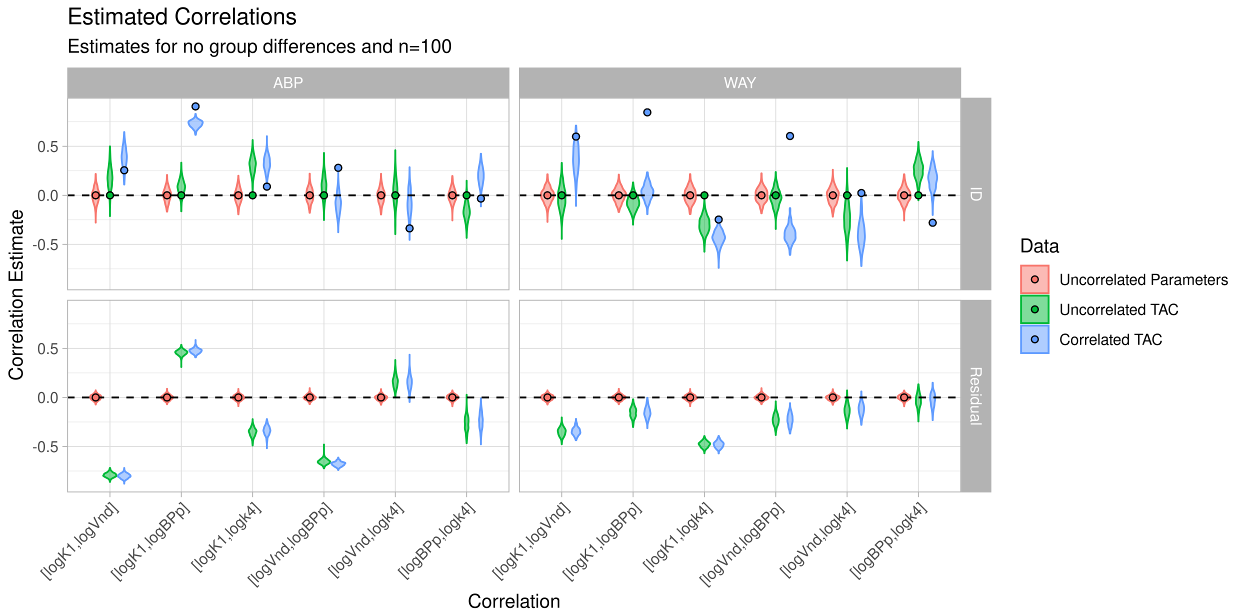

Figure 8: Estimated correlations in simulated data for sample sizes of 100 individuals per group. True values are depicted by points. Note that there no true values for the residual correlations in the TAC simulations, as the residual variance consists of a combination of individual x region variation and TAC fitting error

In the individual (τ) correlation matrices, there were clear differences between the estimated correlation matrices for the correlated and uncorrelated datasets for each tracer, suggesting that the correlation structure of the data does affect the estimated multivariate correlation structure even if the recovery of the individual correlation coefficients themselves is poor. As shown below, in uncorrelated data relative to correlated data, false positive rates are unchanged, however power is reduced and standard error and standard deviation are increased.

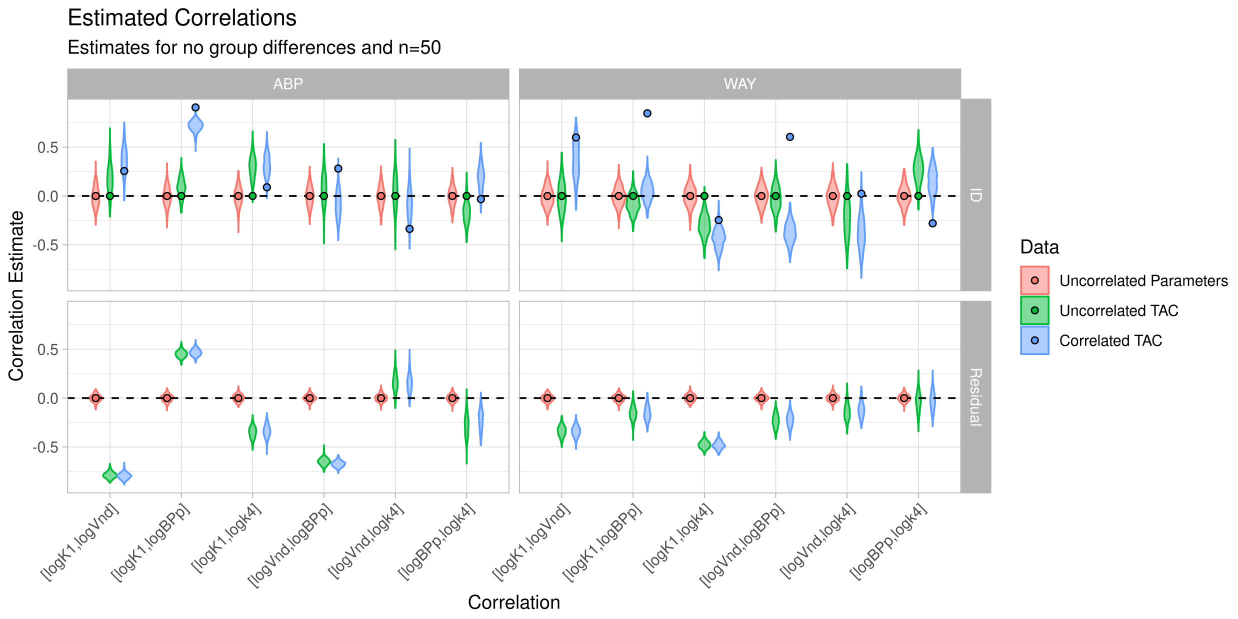

Figure 9: Estimated correlations in simulated data for sample sizes of 50 individuals per group. True values are depicted by points. Note that there no true values for the residual correlations in the TAC simulations, as the residual variance consists of a combination of individual x region variation and TAC fitting error

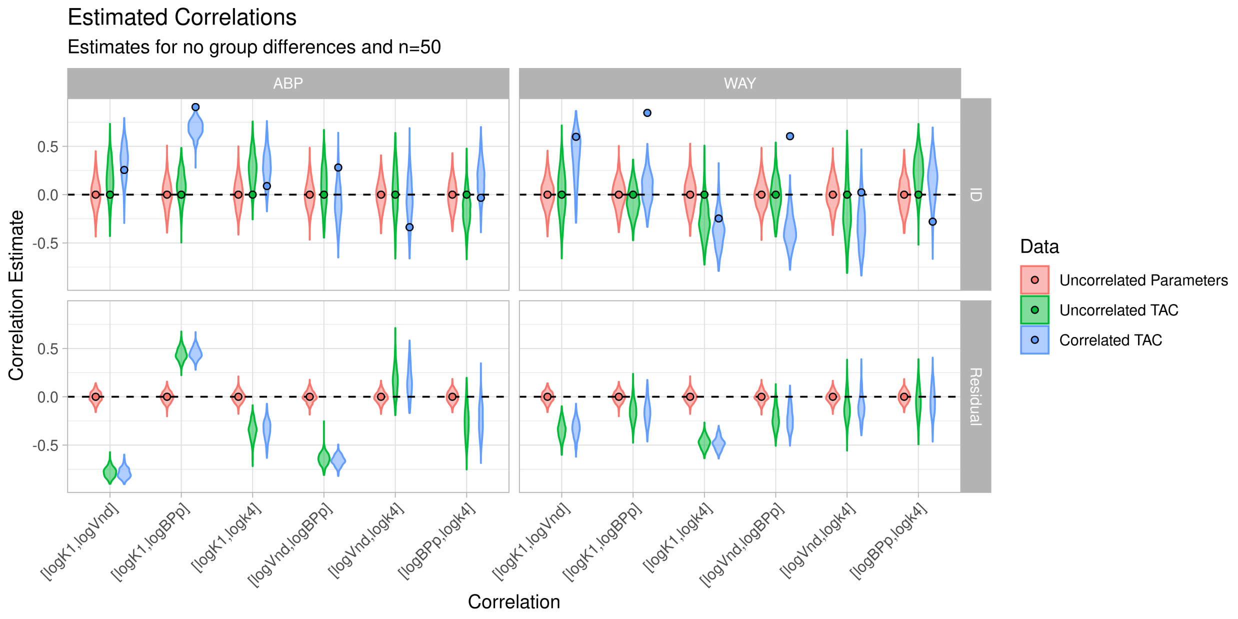

Figure 10: Estimated correlations in simulated data for sample sizes of 20 individuals per group. True values are depicted by points. Note that there no true values for the residual correlations in the TAC simulations, as the residual variance consists of a combination of individual x region variation and TAC fitting error

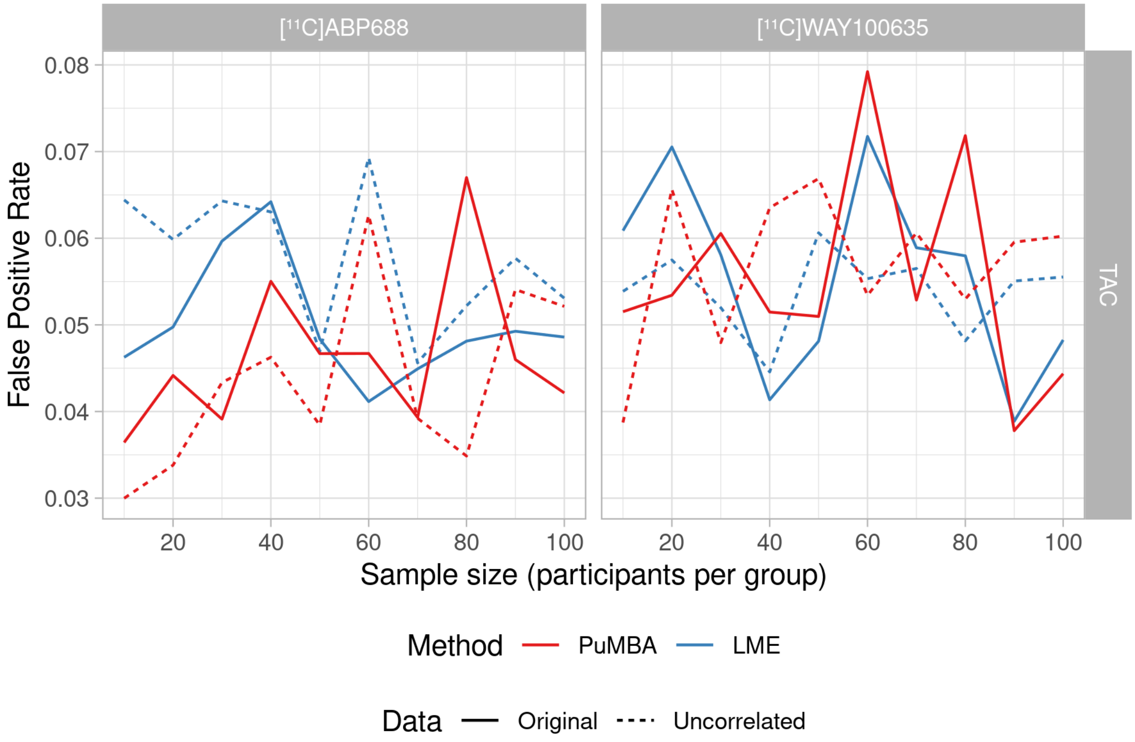

Figure 11: False positive rate across simulated datasets.

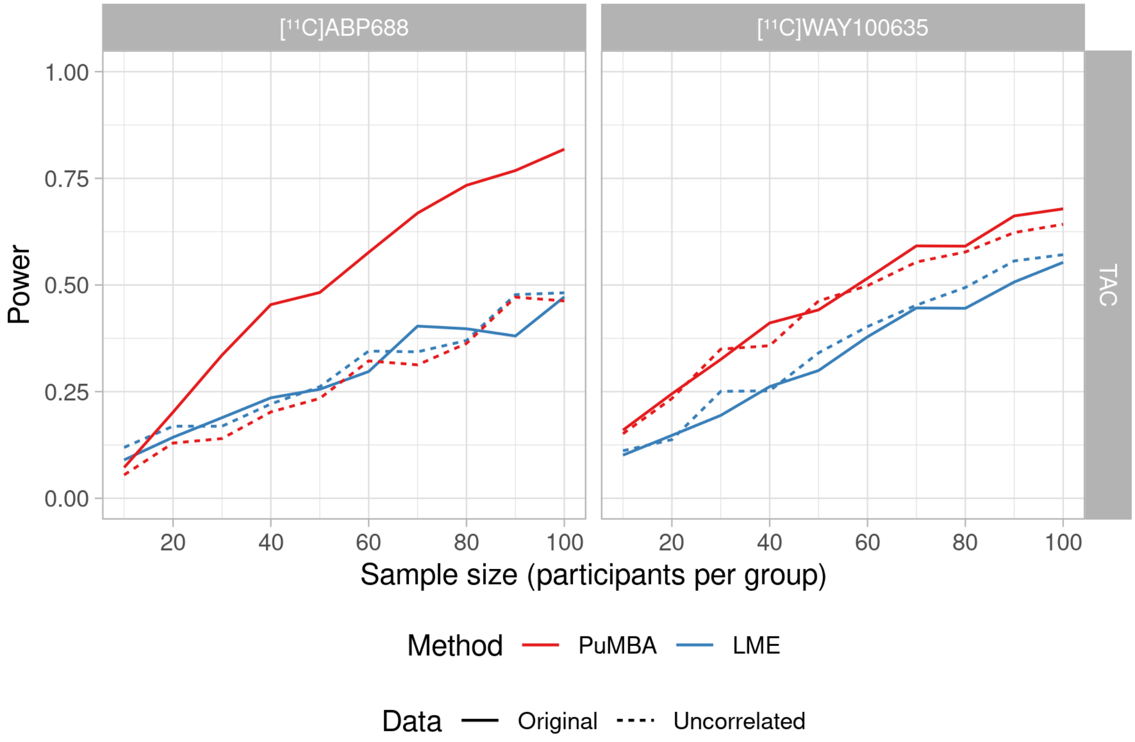

Figure 12: Power across simulated datasets.

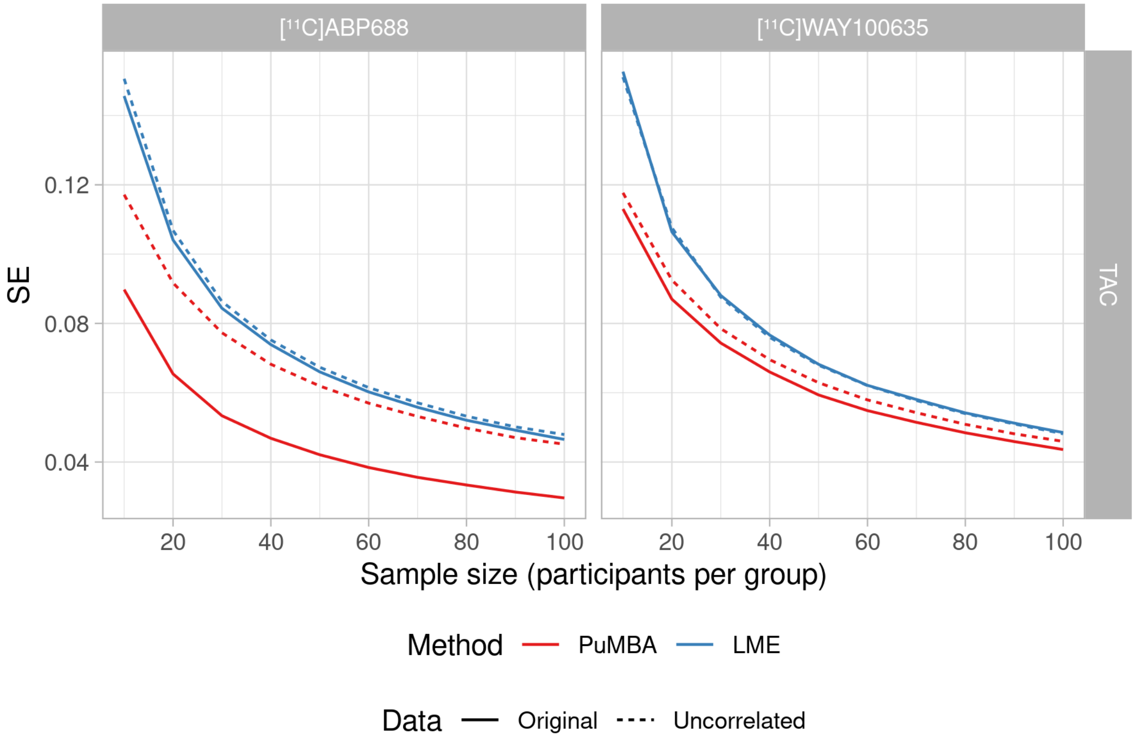

Figure 13: Mean standard error across simulated datasets.

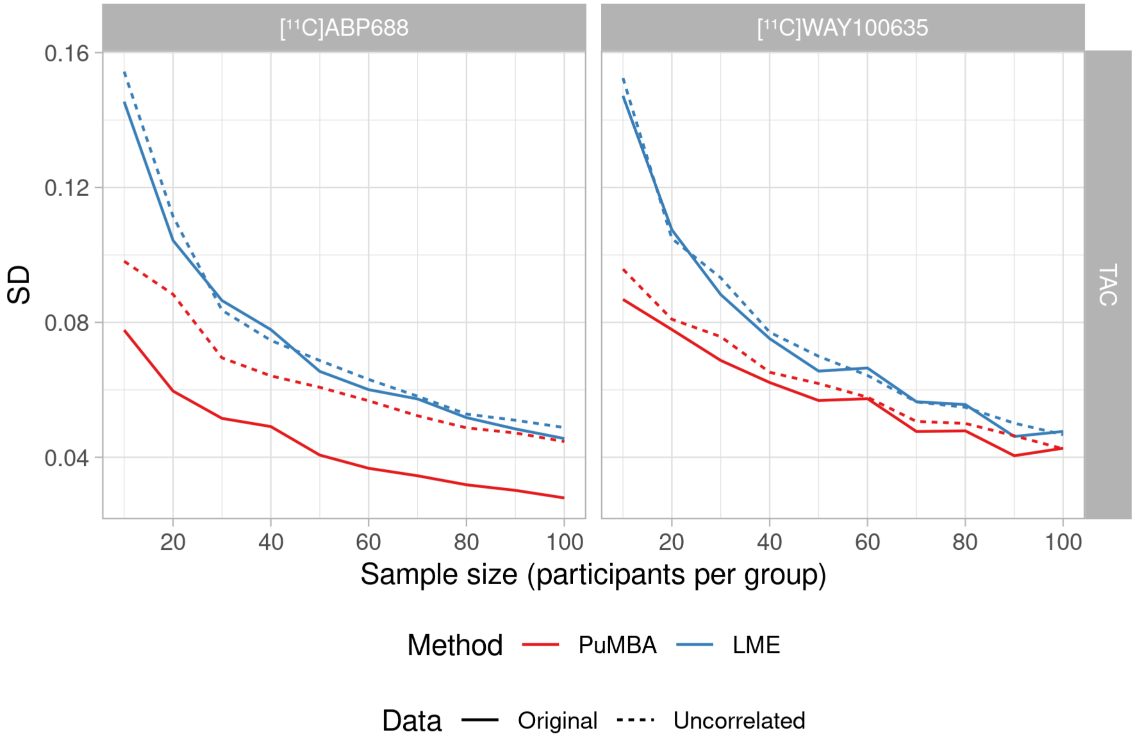

Figure 14: Standard deviation of estimated differences across simulated datasets.

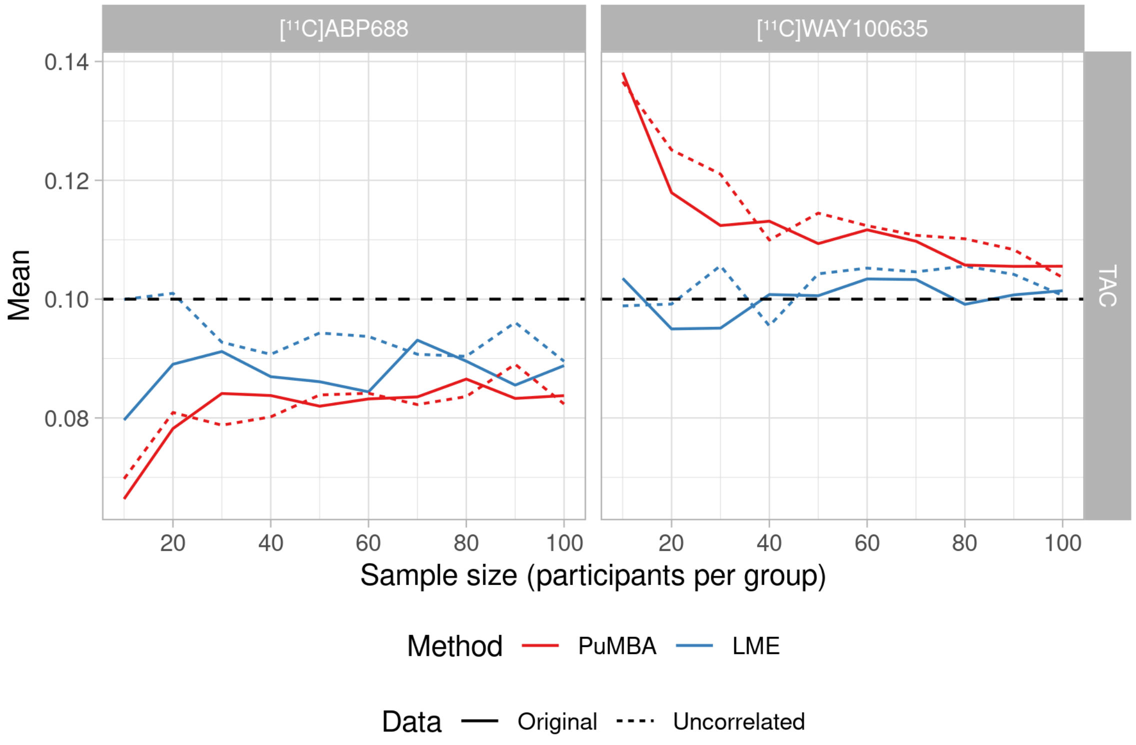

Figure 15: Mean estimated differences across simulated datasets.

Supplementary Materials S6 : Parameter Simulation Additional Figures

Here we show the results of prior misspecification, in which for each simulated dataset, the mean of each global intercept prior was either doubled or halved. Hence, in one dataset, the V_{ND} prior might be centred on a value which is double the original prior, and the K_1 prior might be centred on a value which is half of the original prior mean value. This does not seem to make an appreciable impact on the results.

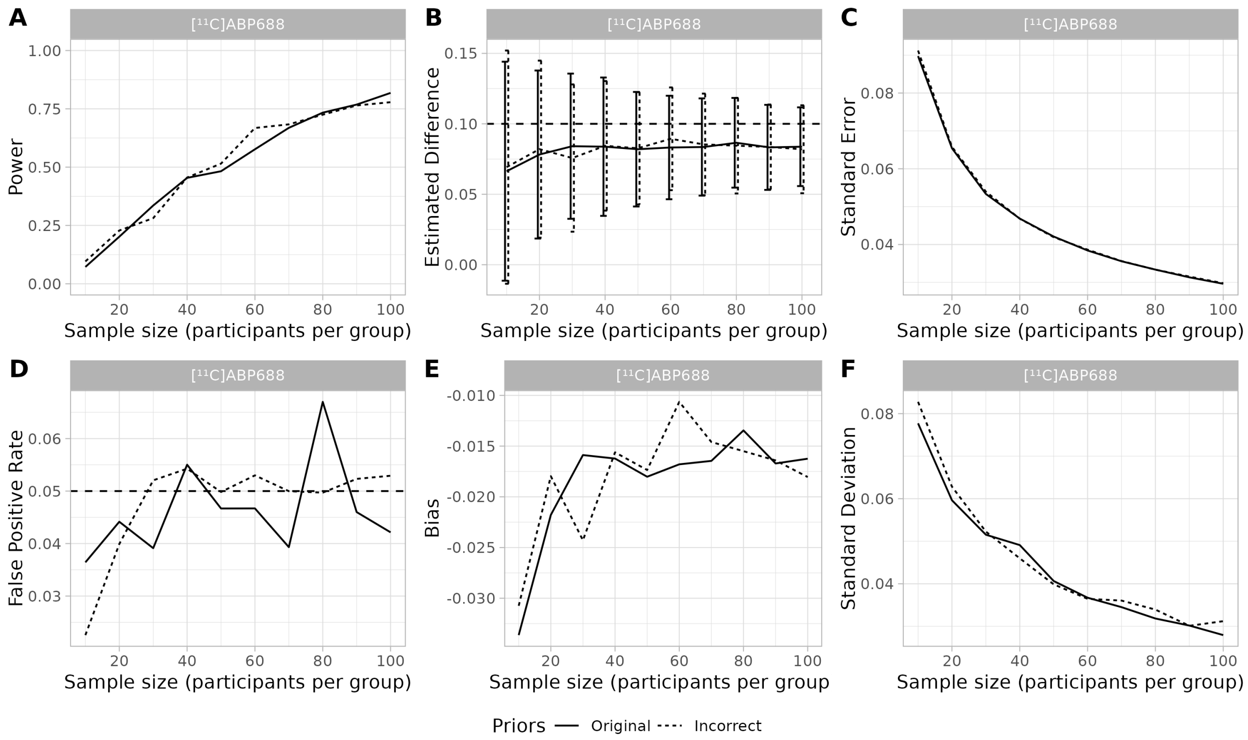

Figure 16: The influence of misspecification of the global intercept priors on the evaluated matrics.

Supplementary Materials S7 : Parameter Simulation Additional Figures

Here we show the additional figures regarding the outcomes of the parameter simulations, representing the false positive rate, the mean estimated differences and, the standard deviation across datasets. Please refer to the manuscript for how each of these metrics are defined.

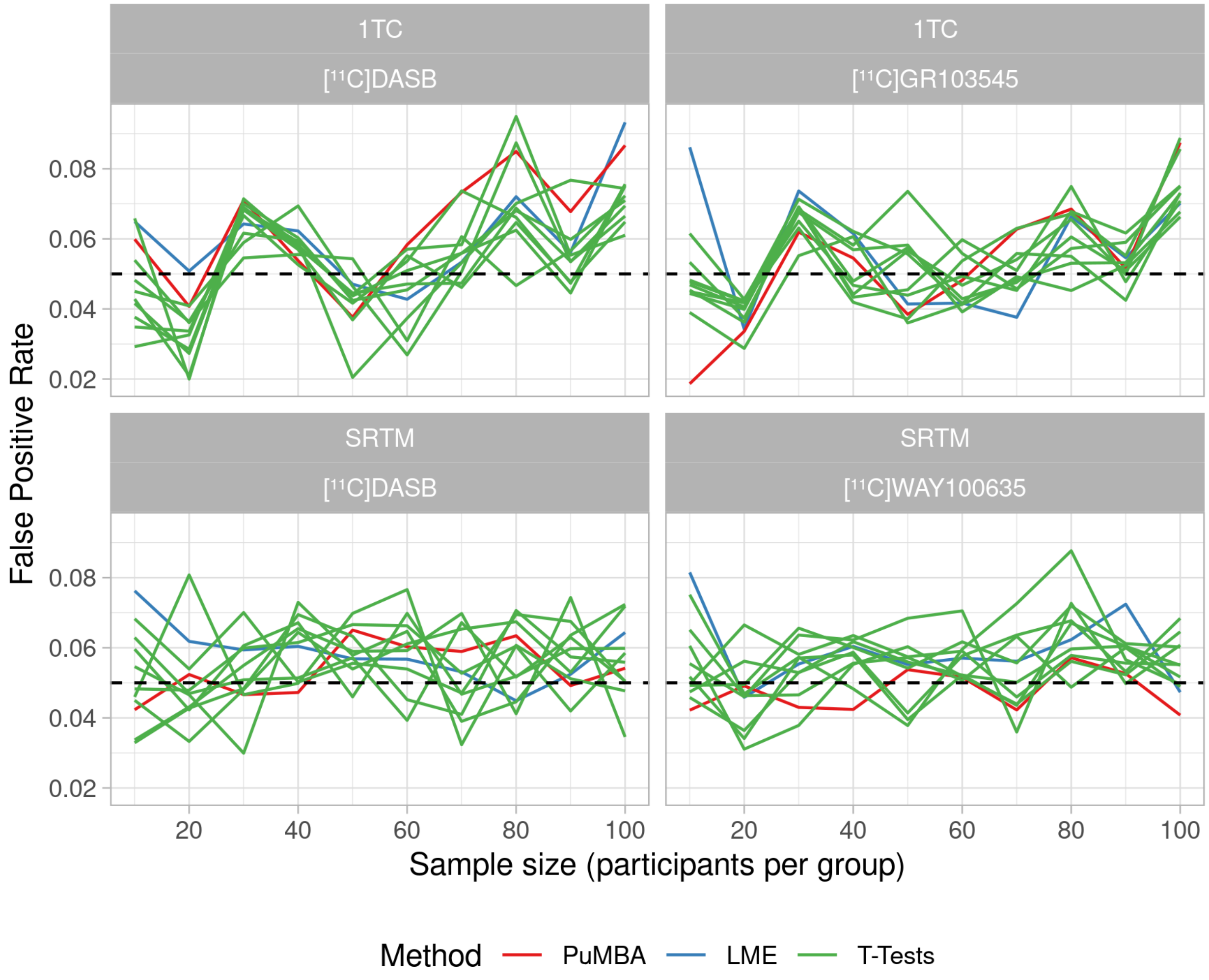

Figure 17: False positive rate across simulated datasets.

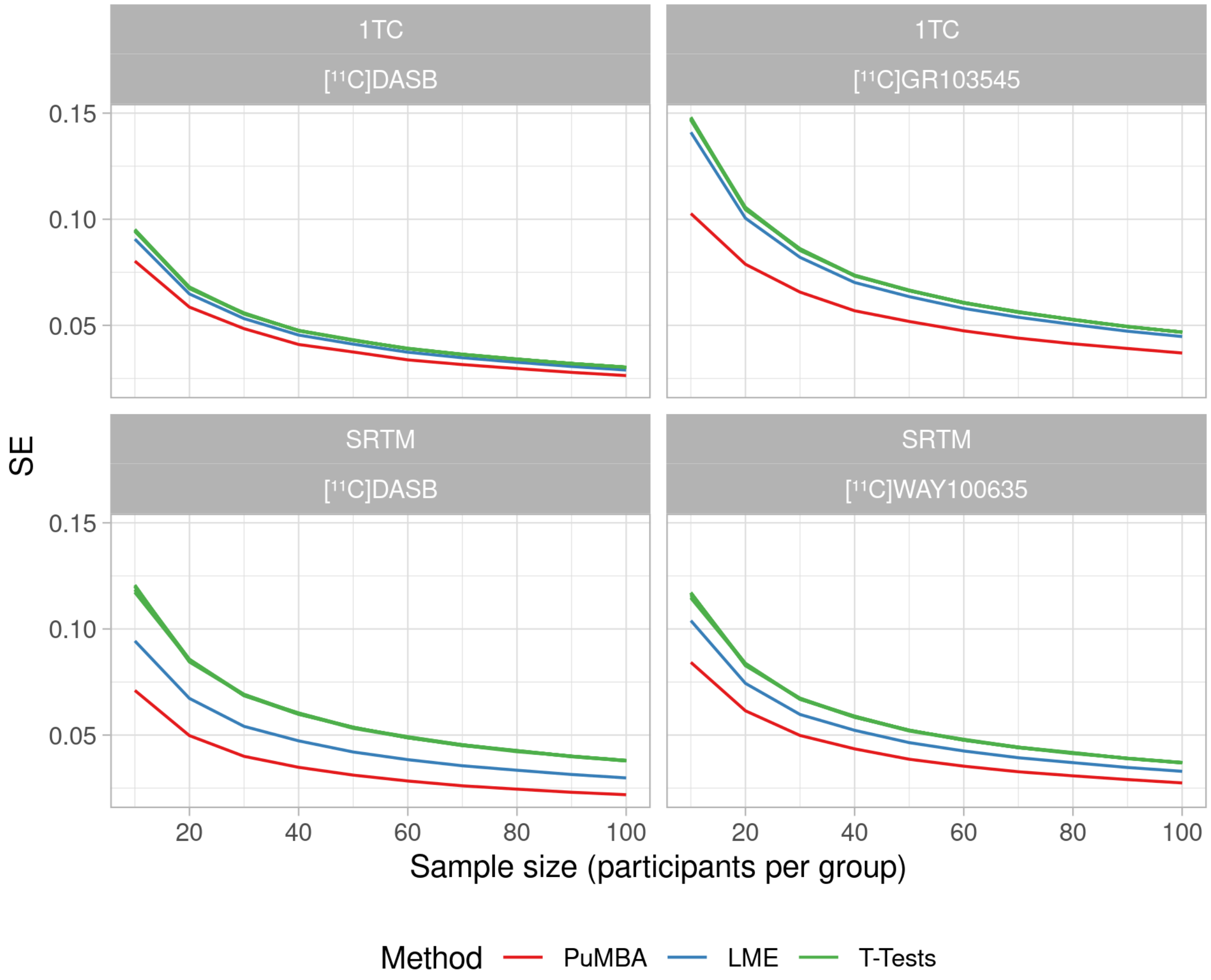

Figure 18: Mean standard error across simulated datasets.

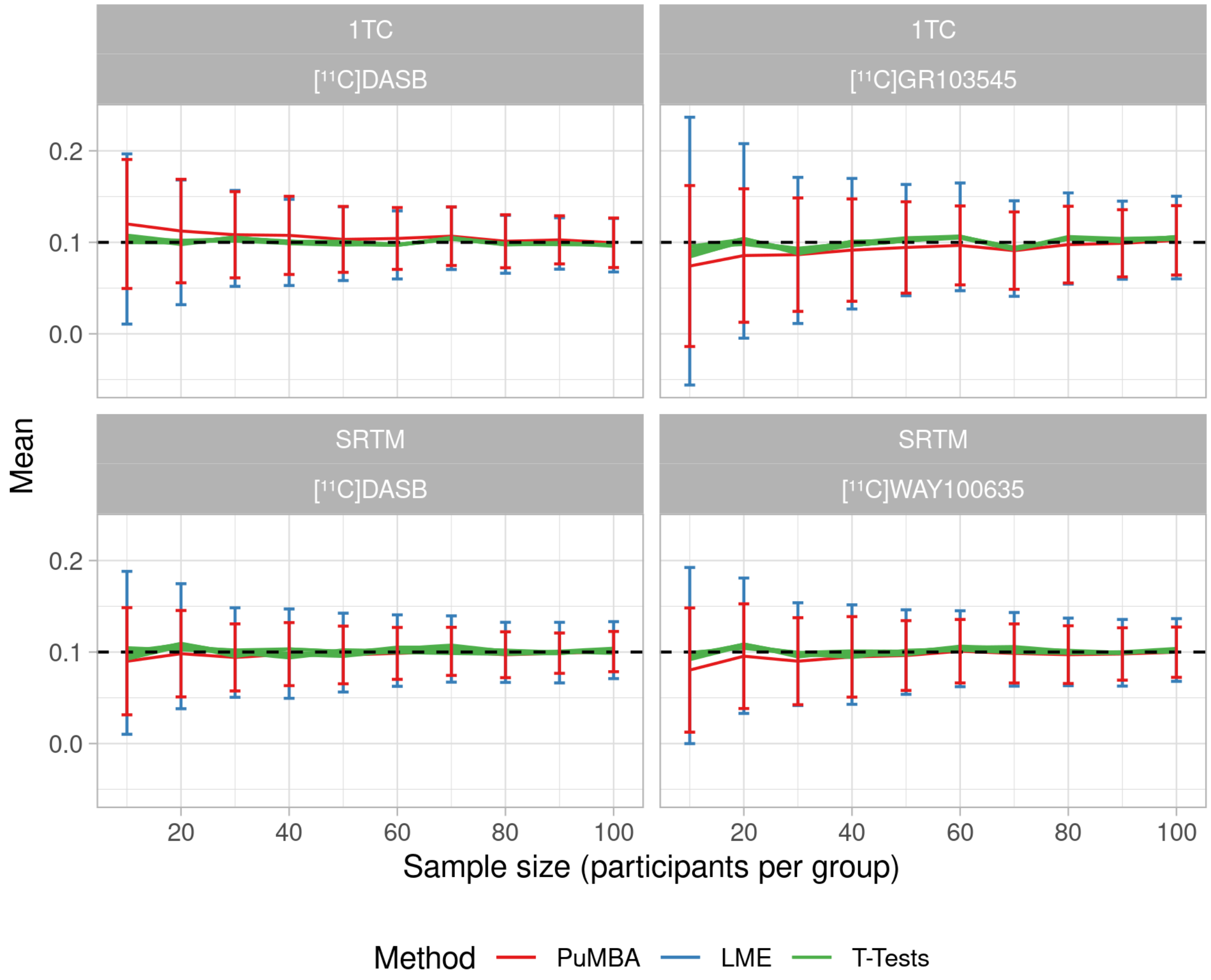

Figure 19: Mean estimated differences across simulated datasets.

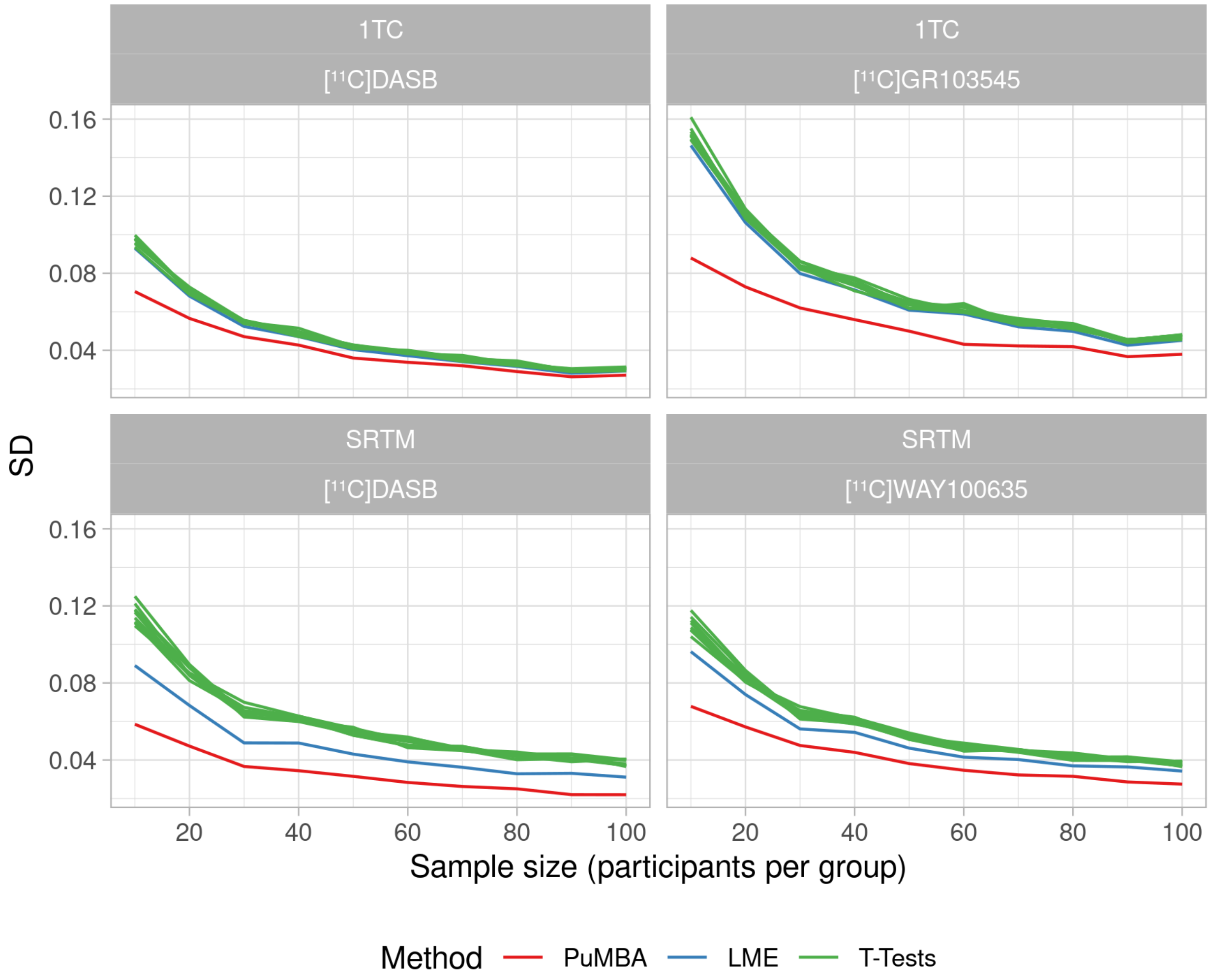

Figure 20: Standard deviation of estimated differences across simulated datasets.
